# Frequency Shapes the Quality of Tactile Percepts Evoked through Electrical Stimulation of the Nerves

**DOI:** 10.1523/JNEUROSCI.1494-21.2021

**Published:** 2022-03-09

**Authors:** Emily L. Graczyk, Breanne P. Christie, Qinpu He, Dustin J. Tyler, Sliman J. Bensmaia

**Affiliations:** ^1^Department of Biomedical Engineering, Case Western Reserve University, Cleveland, Ohio 44106; ^2^Louis Stokes Cleveland Department of Veterans Affairs Medical Center, Cleveland, Ohio 44106; ^3^Research and Exploratory Development Department, Johns Hopkins University Applied Physics Laboratory, Laurel, Maryland 20723; ^4^Committee on Computational Neuroscience, University of Chicago, Chicago, Illinois 60637; ^5^Department of Organismal Biology and Anatomy, University of Chicago, Chicago, Illinois 60637

**Keywords:** amputee, artificial touch, neural interfaces, psychophysics, temporal code, touch

## Abstract

Electrical stimulation of the peripheral nerves of human participants provides a unique opportunity to study the neural determinants of perceptual quality using a causal manipulation. A major challenge in the study of neural coding of touch has been to isolate the role of spike timing—at the scale of milliseconds or tens of milliseconds—in shaping the sensory experience. In the present study, we address this question by systematically varying the pulse frequency (PF) of electrical stimulation pulse trains delivered to the peripheral nerves of seven participants with upper and lower extremity limb loss via chronically implanted neural interfaces. We find that increases in PF lead to systematic increases in perceived frequency, up to ∼50 Hz, at which point further changes in PF have little to no impact on sensory quality. Above this transition frequency, ratings of perceived frequency level off, the ability to discriminate changes in PF is abolished, and verbal descriptors selected to characterize the sensation change abruptly. We conclude that sensation quality is shaped by temporal patterns of neural activation, even if these patterns are imposed on a fixed neural population, but this temporal patterning can only be resolved up to ∼50 Hz. These findings highlight the importance of spike timing in shaping the quality of a sensation and will contribute to the development of encoding strategies for conveying touch feedback through bionic hands and feet.

**SIGNIFICANCE STATEMENT** A major challenge in the study of neural coding of touch has been to understand how temporal patterns in neuronal responses shape the sensory experience. We address this question by varying the pulse frequency (PF) of electrical pulse trains delivered through implanted nerve interfaces in seven amputees. We concomitantly vary pulse width to separate the effect of changing PF on sensory quality from its effect on perceived magnitude. We find that increases in PF lead to increases in perceived frequency, a qualitative dimension, up to ∼50 Hz, beyond which changes in PF have little impact on quality. We conclude that temporal patterning in the neuronal response can shape quality and discuss the implications for restoring touch via neural interfaces.

## Introduction

Physical interactions with our environment give rise to a rich and complex tactile experience. While a touch can be readily localized to a specific location on the body and described as light or strong, its sensory quality is multifaceted and often difficult to describe. For example, the tactile experience of a fabric is different from that of a vibration. Sensory quality does not fall on one continuum or even a few. Indeed, even a single sensory subspace, such as that for tactile texture, is complex and can be further broken down into component dimensions and subspaces (Hollins et al., 1993, 2000; Bergmann Tiest and Kappers, 2006; Lieber and Bensmaia, 2019).

The neural underpinnings of sensory quality are also difficult to identify. The perceived location of a touch is determined by the receptive field location of the activated neural population (Ochoa and Torebjörk, 1983; Schady et al., 1983; Johansson and Flanagan, 2009). The magnitude of a percept is determined by the overall population spike rate evoked in the peripheral nervous system (Muniak et al., 2007; Graczyk et al., 2016). Quality, on the other hand, is determined by the specific spatiotemporal pattern of activation elicited in the nervous system. Some patterns of activation give rise to a texture percept, others to a motion percept, and these patterns can co-occur and be multiplexed in the nerve (Goodman and Bensmaia, 2018).

One determinant of quality is the degree to which each class of tactile nerve fibers is activated by the stimulus. Indeed, the sensation evoked when an individual nerve fiber is electrically activated depends on its afferent class—whether it is slowly adapting or rapidly adapting, type I or type II (Ochoa and Torebjörk, 1983; Torebjörk et al., 1987). As a result, one reason different tactile stimuli evoke sensations of different qualities is because they differentially activate different classes of tactile afferent fibers. For example, skin vibrations recruit afferent populations in a frequency-dependent manner (Talbot et al., 1968; Freeman and Johnson, 1982; McGlone and Reilly, 2010). This differential recruitment accounts in part for the frequency dependence of vibrotactile pitch, the qualitative dimension associated with changes in vibratory frequency (Goff, 1967; Roy and Hollins, 1998; McGlone and Reilly, 2010).

Another determinant of quality is the temporal patterning in the spiking response. For example, the perception of texture is shaped by the millisecond-level timing of sequences of spikes evoked in nerve fibers (Weber et al., 2013). Similarly, phase-locked responses of nerve fibers to skin vibrations underlie their perceived vibrotactile pitch, which complements the information about frequency carried in the pattern of recruitment across classes (Mackevicius et al., 2012; Birznieks and Vickery, 2017). In natural mechanical touch, the co-occurrence of these coding principles—differential recruitment and temporal patterning—makes it difficult to disentangle their respective roles in the determination of sensory quality.

Electrical stimulation offers a unique opportunity to examine the contribution of spike timing on tactile perception independently of the relative recruitment of afferent classes. An electrical pulse train delivered to the nerve will synchronously activate local neurons regardless of fiber class, as there are no known class-specific properties to enable preferential recruitment (Mogyoros et al., 1996; Howells et al., 2012). The impact of stimulation frequency on quality is thus mediated by the phase-locked spiking response, as all fiber classes are equally likely to be activated. Thus, by varying stimulation frequency in the peripheral nerve, we can directly examine the role of spike timing on quality perception.

Electrical stimulation of somatosensory peripheral nerves evokes vivid tactile sensations that are experienced on the hand (Dhillon and Horch, 2005; Ortiz-Catalan et al., 2014; Raspopovic et al., 2014; Tan et al., 2014; Davis et al., 2016). While increasing stimulation frequency in the peripheral nerve is known to increase the perceived intensity of the percept, mediated by a concomitant increase in the population firing rate (Graczyk et al., 2016), the effect on quality is less clear. To fill this gap, we sought to understand how temporal patterns of activation in the nerve affect the quality of tactile percepts. We approached this question in two sets of psychophysical experiments with human amputees implanted with multichannel nerve cuff electrodes. First, we investigated how changes in the frequency of electrical stimulation impact the perceived frequency of the stimulus. In these experiments, participants judged the percept evoked by a pulse train along a single dimension or discriminated pulse trains that varied in frequency. Second, we investigated the impact of pulse frequency (PF) on a multidimensional space defined by verbal descriptors to achieve a more holistic understanding of how pulse frequency shapes the quality of an electrically evoked percept. Understanding how spike timing shapes quality will help unravel the peripheral neural code of touch and will lead to the development of approaches to convey a richer, more natural touch experience to individuals with bionic hands and feet.

## Materials and Methods

### Participants

Seven people, all male, volunteered for this study. Four participants had unilateral acquired amputations of the upper limb below the elbow, and three had unilateral acquired amputations of the lower limb below the knee. All participants were implanted with multicontact peripheral nerve cuff electrodes, either flat interface nerve electrodes (FINEs) or composite FINEs (C-FINEs; Freeberg et al., 2017, Fig. 1*a*). Upper limb participants (referred to as UL01-UL04) were implanted with 8-channel FINEs or 16-channel C-FINEs around the median, ulnar, and/or radial nerves between 3 and 7 years before participation in the present study. Contacts were selected for testing that elicited comfortable sensations on the anterior surface of the hand. Lower limb participants (LL01-LL03) were implanted with 16-channel C-FINEs around the sciatic and tibial nerves between 1 and 3 years before participation in the present study. Contacts were selected for testing that elicited comfortable sensations on the plantar surface of the foot or posterior aspect of the ankle. Percutaneous leads connected the cuff electrodes to an external neurostimulator. Stimulation waveforms were square, biphasic, cathodic first, and charge balanced. Stimulation parameters were set in MATLAB (MathWorks) and then sent to a computer running xPC Target (MathWorks), which then drove the stimulator. The stimulator could produce stimulation with pulse width (PW) in the range of 5–255 µs in increments of 1 µs, pulse amplitude (PA) in the range of 0–2 mA in increments of 0.1 mA, and interpulse intervals in the range of 1–10,000 ms in increments of 1 ms.

The Louis Stokes Cleveland Department of Veterans Affairs Medical Center Institutional Review Board and Department of the Navy Human Research Protection Program approved all procedures. This study was conducted under an Investigational Device Exemption obtained from the U.S. Food and Drug Administration. All participants provided written informed consent to participate in this study, which was designed in accordance with relevant guidelines and regulations.

### Frequency estimation task

**Figure 1.**
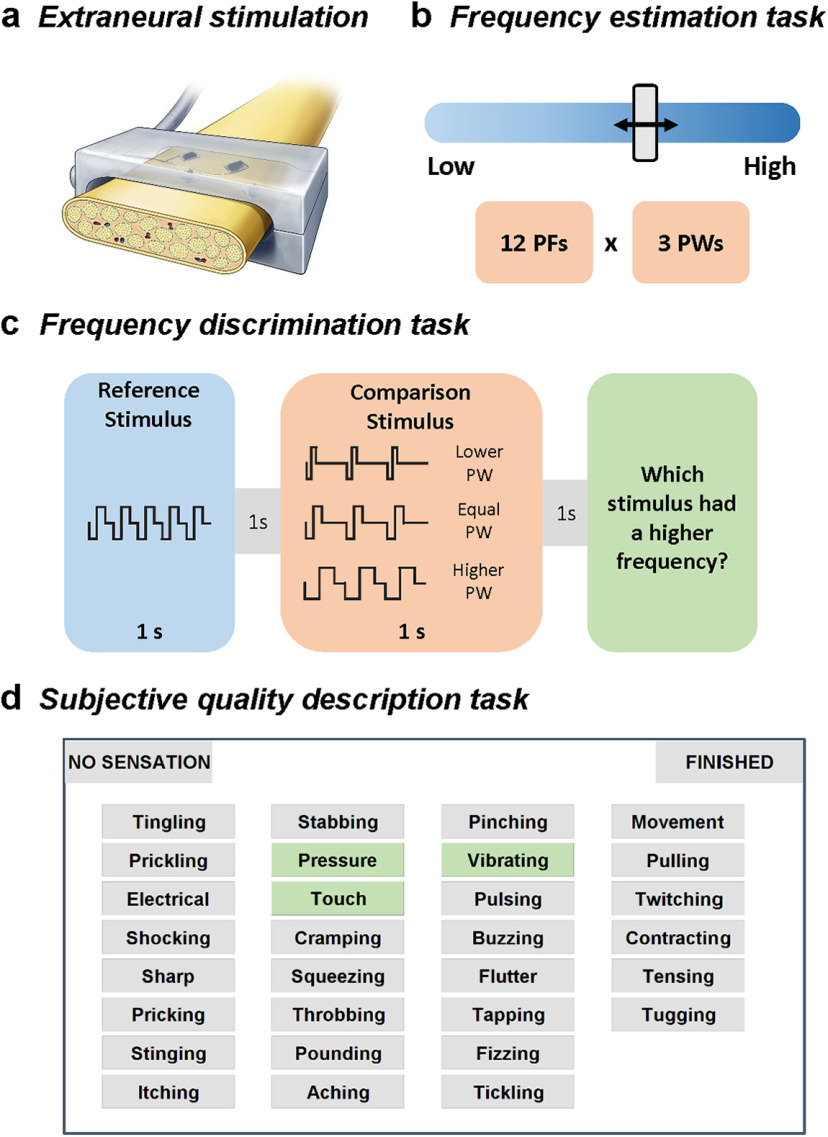
Experimental design. ***a***, Electrical stimulation was delivered by an external neurostimulator through percutaneous leads to FINEs or C-FINEs implanted on the residual peripheral nerves of four transradial and three transtibial amputees. Stimulation consisted of trains of square, biphasic, charge-balanced pulses. ***b***, Frequency estimation task. Three participants were asked to estimate the perceived frequency of an evoked percept by moving a slider along a horizontal bar. Stimuli varied in both stimulation PF and PW. ***c***, Frequency discrimination task. Four participants judged which of two sequentially presented pulse trains was higher in perceived frequency, while ignoring any differences in perceived intensity. ***d***, Quality description task. Six participants selected sets of words that described the quality of the evoked percept. Participants could select as many words as they wished to describe each stimulus.

In each trial, a 1 s pulse train was delivered, and participants estimated the perceived frequency of the evoked sensation. Estimations were made using a visual analog scale displayed on a computer monitor, which displayed “slowest possible frequency” on the far left of the scale and “fastest possible frequency” on the far right of the scale (Fig. 1*b*). Participants were asked to ignore changes in intensity and location when making frequency estimates. A 3 s intertrial interval was enforced to minimize the effects of perceptual adaptation (Graczyk et al., 2018).

For each electrode contact, we tested 12 PFs and 3 PWs. PFs were set to 2, 5, 10, 20, 35, 50, 65, 80, 100, 200, 500, and 1000 Hz. The three PWs were chosen on a per-contact basis to span the full range of suprathreshold, comfortable intensities. Briefly, we used a staircase procedure to find the minimum PA value that produced a detectable percept at the maximum PW of the stimulator (255 µs). This PA value was typically between 0.5 and 1 mA. We then used a two-alternative forced-choice tracking paradigm to find the minimum PW value that elicited a detectable percept at this threshold PA (Graczyk et al., 2016). The “low” PW value in the estimation task was the minimum PW that was reliably perceived at 2 Hz. The “high” PW value was chosen as the maximum PW that was comfortable at 500 Hz. The “mid” PW value was chosen as the midpoint between the low and high values. Preliminary tests with each contact indicated that these PWs resulted in clear differences in perceived intensity. The 36 stimuli were presented in pseudorandom order 15 times/contact, split into six experimental blocks of 90 trials, all performed on the same day. Five electrode contacts across three participants were included in this analysis.

Frequency ratings were normalized by dividing each estimate by the within-block mean rating. Normalized ratings were then averaged within contact for each PW and PF condition. We then fit piecewise linear functions to the base-10 logarithm of frequency, separated by PW condition. For each fit, coefficient estimates over their relevant ranges were randomly generated 10 times, and the combination with the lowest mean squared error was selected. The transition between the rising and falling phases of the perceived frequency ratings was obtained from the optimized parameters. The transition metric was averaged across participants, contacts, and PWs after first removing two values derived from poor fits (*R*^2^ < 0.5) and one outlier that was more than 2 SDs above the mean. Means and SEs of the frequency estimates were then calculated across participants and contacts within each PW and PF condition.

### Frequency discrimination task

Each trial consisted of two successive 1 s pulse trains separated by a 1 s interstimulus interval. Participants judged which of the two sequentially presented pulse trains was higher in perceived frequency. In each experimental block, a standard stimulus (at 20, 50, or 100 Hz) was paired with a comparison stimulus whose PF and PW varied from trial to trial (Fig. 1*c*). The comparison PFs ranged from 25% to 175% of the standard PF. Specifically, for the 20 Hz reference, the comparison PFs were 5, 10, 16, 18, 20, 22.4, 25, 30, and 35 Hz; for the 50 Hz reference, the comparison PFs were 12.5, 25, 40, 45, 50, 55.55, 62.5, 76, and 90.9 Hz; and for the 100 Hz reference, the comparison PFs were 25, 50, 83, 90, 100, 111, 125, 145, and 166 Hz. The comparison PW took on one of the following three values: one shorter than, one equal to, and one longer than the standard PW. PWs were based on the same criteria as in the frequency estimation task. All PW values were between 70% and 130% of the standard PW. The standard stimulus was always at the intermediate PW. In each block, each stimulus pair was presented 10 times, and both the order of stimuli within the pair and the order of the pairs varied pseudorandomly. The intertrial interval was enforced to be at least 3 s long. Thus, each experimental block evaluated one electrode contact and consisted of 270 trials (9 comparison PFs × 3 comparison PWs × 10 repetitions). Eight contacts across four participants, six contacts across two participants, and six contacts across two participants were evaluated for the 20, 50, and 100 Hz standard, respectively.

Psychophysical performance was calculated as the proportion of trials in which the comparison stimulus was judged to be higher in perceived frequency than the standard stimulus. Psychometric curves were then constructed by fitting a cumulative normal density function to these proportions. Separate psychometric functions were fit for each standard frequency (20, 50, and 100 Hz). Just noticeable differences (JNDs) were calculated as the change in frequency required to achieve 75% correct performance. Two JND estimates were obtained from each psychometric function, one above and one below the standard, and these two estimates were averaged. Sessions in which performance did not achieve the 75% threshold performance criterion (above or below the standard) were considered to have an undefined or incalculable JND and were considered to be “poor” performance contacts. These data points were omitted from further analysis. The point of subjective equality (PSE) was defined as the comparison frequency that corresponded to 50% correct performance, as calculated from the fitted psychometric functions.

### Subjective quality description task

On each trial, a 2 s pulse train was delivered and participants were asked to indicate which subset of a list of 30 qualitative descriptors applied to the percept (Fig. 1*d*, word list). These words were selected based on prior studies of language associated with natural and artificial somatosensation (Adrian, 1919; Goff, 1967; Tashiro and Higashiyama, 1981; Macefield et al., 1990; Kaczmarek and Haase, 2003; Guest et al., 2011; Geng et al., 2012; Perovic et al., 2013). Participants were encouraged to select as many words as necessary to fully describe the sensation. Participants were instructed to use their own definitions for each descriptor, but to maintain consistency throughout each experimental session. For example, if two stimuli felt identical, they were asked to select the same subset of words for both trials. Stimulation was applied at one of nine PFs and three PWs for each stimulus. Stimulation PF was set to 2, 5, 10, 20, 50, 100, 200, 500, and 1000 Hz, and PWs were selected in the same manner as in the frequency estimation task. Each stimulus was presented 20 times in pseudorandomized order. A 2 s intertrial interval was enforced. A “no sensation” option was provided for trials in which the participant did not feel the stimulus. Eleven electrode contacts were tested across all six participants. Stimulation conditions in which no sensation was selected in more than five trials were excluded from the analysis. We computed the proportion of times each descriptor word was selected for each stimulation condition for each contact after no sensation trials were removed. The result was a set of 30 proportions for each stimulation condition.

To compute within-participant consistency in word selection, we ran a correlation analysis of word proportions between the sets of contacts tested within the same participant. Those participants who only performed the subjective quality test with a single contact were excluded from this analysis. To calculate across-participant consistency in word selection, we first averaged the proportions across contacts for each participant, then computed the correlation in these proportions for each pairing of participants.

### Dissimilarity matrices

Having observed that different participants used different words to describe the electrically evoked sensations, we hypothesized that we could achieve a more generalizable representation of tactile quality by computing differences in descriptors between stimulation conditions. To reduce redundant or highly correlated descriptors within each dataset, we ran a principal component analysis (PCA) on the word proportion vectors across all stimuli (a 30 × 27 data matrix) on a participant-by-participant basis and retained the components necessary to explain 95% of the variance. We then projected the stimuli onto this reduced dimensionality space and calculated the pairwise distances between them, resulting in a 27 × 27 distance matrix for each tested electrode contact (*n* = 11). To remove the influence of the number of dimensions on calculated distances, we normalized distances to the maximum intercondition distance (which was set to 1). For some analyses (see Fig. 6*a*), we averaged the coordinates for each PF across PWs and then recomputed the distances, yielding a 9 × 9 distance matrix.

To assess the within-participant consistency in dissimilarities, we computed the correlation between distance matrices obtained from different contacts for each participant. To assess across-participant consistency in dissimilarities, we first averaged the dissimilarities across contacts for each participant then computed the correlation in the dissimilarity matrices for each pair of participants.

To examine the quality dissimilarity between successive PF conditions, we computed the distance between the coordinates of two adjacent PFs in the reduced dimensionality space, then averaged these distances across PWs and contacts.

Finally, we performed a multivariate regression analysis with ΔPF, ΔPW, and their interaction as factors. We performed this analysis after averaging the 27 × 27 distance matrices across participants and contacts. The ΔPF and ΔPW factors were *z* scored before running the regression to standardize the regression coefficients. This analysis was also performed with the data split into the following two sets: one for PFs at <50 Hz and one for PFs at >50 Hz. We verified that the regression model fit to the dissimilarity matrix averaged across contacts and participants yielded the same conclusions as one fit to data pooled across individual contacts and participants. We also fit regression models to individual participants' dissimilarity matrices and verified that the regression coefficients averaged across these models yielded the same conclusions. We then performed a stepwise regression, in which we assessed whether PF accounted for a significant proportion of variance in the dissimilarity ratings after differences in perceived frequency had been regressed out. For this analysis, we first computed differences in perceived frequency for each stimulus pair from the across-contact averages of perceived frequency obtained in the first experiment. We then regressed dissimilarity against differences in perceived frequency ratings and regressed the residuals against *z* scored ΔPF. This analysis was performed separately for stimuli <50 Hz and >50 Hz.

### Statistics

#### Frequency estimation.

We determined the influence of PW on the transition frequency between the rising and falling regimes of perceived frequency using a one-way ANOVA versus PW. We then split the data into the following two regimes: 0–50 Hz and >50 Hz. For each regime, we ran a two-way ANOVA on the normalized frequency estimations pooled across contacts and participants with factors of PW and PF. All statistical analyses were performed in Minitab (Minitab, LLC) with an α level of 0.05.

#### Frequency discrimination.

We studied the influence of PW on the JNDs and PSEs of the psychometric functions using repeated-measures ANOVA with different PWs and different contacts as factors. JNDs and Weber fractions at the 20 Hz standard were compared with their counterparts at 50 Hz using a two-sample *t* test. All statistical analyses were performed in MATLAB with α level of 0.05.

#### Subjective quality reports.

We ran a linear regression to determine how the number of words selected by each participant changed with PF. We compared within- and across-participant correlations in quality descriptors and dissimilarities using a one-way ANOVA with Tukey's pairwise comparisons. We compared quality dissimilarities between successive PFs using a two-way ANOVA with Tukey's pairwise comparisons with factors for PF and PW. These analyses, as well as the univariate and multivariate regressions, were performed in MATLAB and/or Minitab with an α level of 0.05.

## Results

Tactile sensations were elicited by delivering electrical pulse trains to the somatosensory nerves, either via FINEs or C-FINEs (Fig. 1*a*). Four unilateral upper limb amputees and three unilateral lower limb amputees participated in the study.

### Perceived frequency increases with pulse frequency up to ∼50 Hz

First, we assessed the degree to which changes in frequency can be judged along a single perceptual continuum. To this end, we delivered stimuli that varied in PF and asked participants to rate the perceived frequency by positioning a slider along a horizontal bar (Fig. 1*b*). To ensure that participants rated frequency rather than intensity, we varied the stimulation PW, which also modulates perceived intensity (Graczyk et al., 2016), from detection threshold to the maximum comfortable level. If frequency estimates were based solely on perceived intensity, then ratings would increase with increases in either PF or PW. PWs were scaled based on the detection threshold, which was measured for each contact separately.

**Figure 2.**
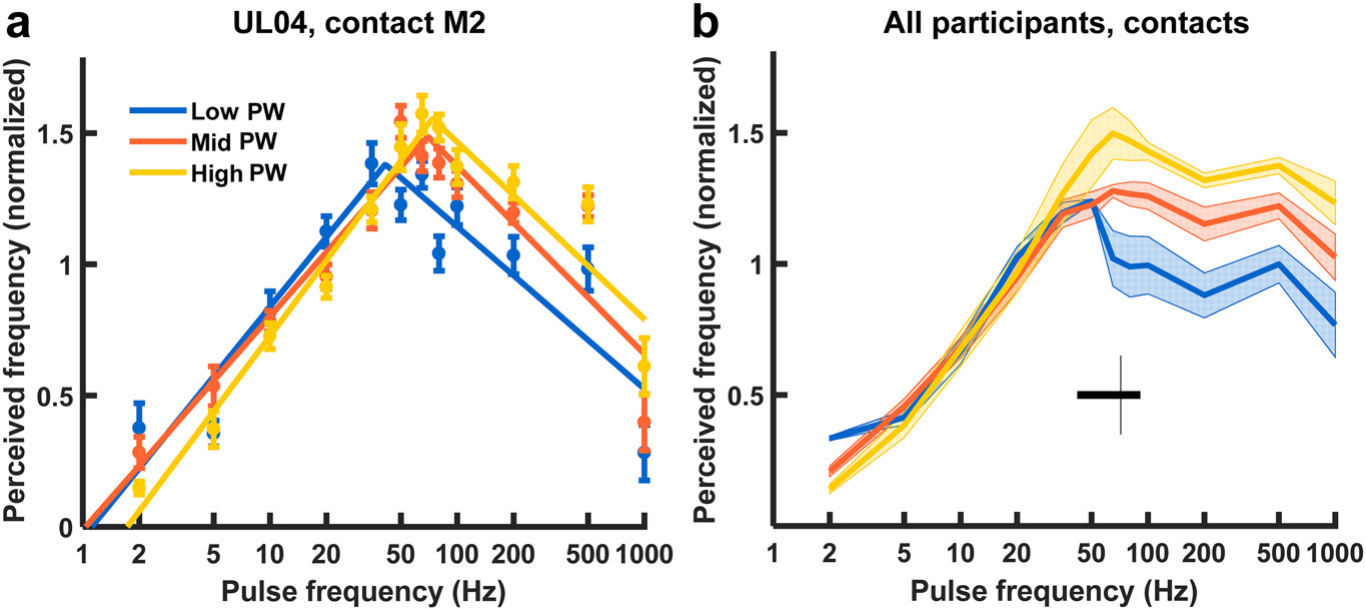
Perceived frequency estimates. ***a***, Perceived frequency versus PF for a representative electrode contact (UL04, median contact M2). Filled circles denote average perceived frequency ratings (*n* = 15/condition), normalized to the mean within experimental block (six blocks). Color denotes the PW level, and error bars denote the SEM. Solid lines represent piecewise logarithmic functions fit to the data. ***b***, Average perceived frequency across all electrode contacts and participants versus stimulation PF (*n* = 15). Color denotes the PW level, and the shaded region corresponds to the SEM. The boxplot depicts the transition frequency in the piecewise functions across participants and contacts (*n* = 12). The vertical line in the boxplot depicts the median, and the shaded region depicts the interquartile range of transition frequency. Note that the abscissa is logarithmic.

At low PFs, perceived frequency increased logarithmically with PF and was independent of PW (Fig. 2). At higher PFs, perceived frequency remained constant or even decreased with increases in PF and was instead modulated by PW (Fig. 2*b*). We fit piecewise logarithmic functions to the relationships between perceived frequency and PF to determine the transition point between the rising and falling (or sustained) portions of the relationships (*R*^2^ = 0.93 ± 0.01, mean ± SEM; Fig. 2*a*). We found the transition frequency to be 66.0 ± 1.1 Hz (mean ± SEM). Transition frequency was highly consistent across participants and contacts, and was not significantly modulated by PW (ANOVA: *F*_(2,11)_ = 0.43, *p* = 0.66).

Having identified two different regimes in the PF response characteristic, we then examined the impact of PF and PW on perceived frequency in each regime separately. Below the transition PF, perceived frequency was significantly dependent on PF (*F*_(5,82)_ = 27.83, *p* < 0.001) but not PW (*F*_(2,82)_ = 1.58, *p* = 0.212). Above the transition PF, perceived frequency ratings depended on PW (*F*_(2,82)_ = 14.08, *p* < 0.001) but not PF (*F*_(5,82)_ = 1.33, *p* = 0.262). In summary, changes in PF exert a systematic effect on perceived frequency up to ∼60 Hz, and this perceptual effect can be clearly distinguished from perceived magnitude. Increases in PF >60 Hz do not change the quality of the percept along the perceived frequency continuum.

### Frequency discrimination is abolished above ∼50 Hz

Having found that the effects of PF on electrically evoked tactile percepts occupy two regimes—one at low and one at high frequencies—we next examined whether these regimes might be reflected in the participants' ability to discriminate changes in PF. To this end, we asked participants to judge which of two sequentially presented pulse trains was higher in perceived frequency (Fig. 1*c*). In each experimental block, which consisted of 270 trials, a standard stimulus (at 20, 50, or 100 Hz) was paired with a comparison stimulus whose PF and PW varied from trial to trial over a range (25–175% of the standard PF). The comparison PW took on one of the following three values: one shorter than, one equal to, and one longer than the standard PW (scaled based on the threshold PW; see Materials and Methods). The variation in PW was intended to reduce or abolish the informativeness of perceived magnitude, which is modulated by changes in both PW and PF (Graczyk et al., 2016).

#### Discrimination with a constant pulse width

**Figure 3.**
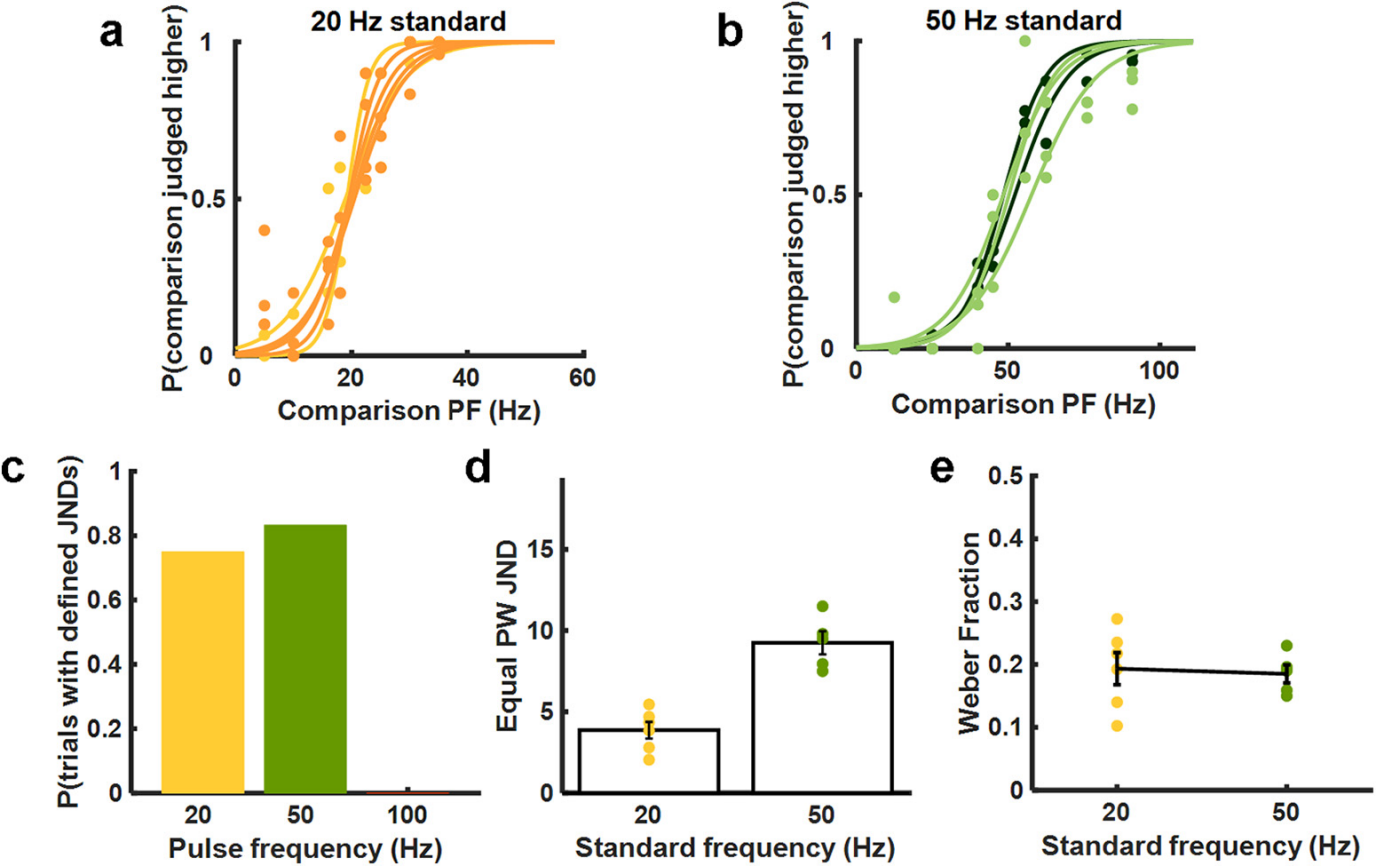
PF discrimination performance with constant PW. ***a***, Performance with the 20 Hz standard for participants UL02 (yellow) and UL04 (orange). Each curve denotes the mean performance with a different electrode contact (*n* = 6). ***b***, Performance with the 50 Hz standard across contacts (*n* = 5) for UL02 (light green) and UL04 (dark green). ***c***, Proportion of stimulation contacts yielding defined JNDs for the three standard frequencies when comparison and standard PWs are equal. Participants achieved threshold performance on 75% (6 of 8) contacts tested with the 20 Hz standard and 83% (5 of 6) contacts tested with the 50 Hz standard. Participants never achieved criterion performance (0 of 7) with the 100 Hz standard. ***d***, JNDs for the equal PW conditions with the 20 Hz (*n* = 6) and 50 Hz (*n* = 5) standards. ***e***, The Weber fractions for the 20 Hz (*n* = 6) and 50 Hz (*n* = 5) standards were statistically indistinguishable.

First, we examined participants' ability to distinguish changes in PF independently of PW by only analyzing same-PW pairs. We found that participants could discriminate on the basis of frequency on most electrode contacts when the standard PF was set at 20 or 50 Hz, as evidenced by smooth psychometric functions over the range tested (Fig. 3*a*,*b*). With the 100 Hz standard, however, PF discrimination performance was very poor on all contacts, and participants never achieved criterion performance (75% correct; Fig. 3*c*; see Materials and Methods). The JND—defined as the difference in PF that yields 75% correct discrimination—was 3.867 ± 0.51 and 9.250 ± 0.71 Hz (mean ± SEM) for the 20 and 50 Hz standards, respectively, yielding nearly constant and statistically indistinguishable Weber fractions (0.193 ± 0.03 and 0.185 ± 0.01 Hz; Fig. 3*d*–*e*). In contrast, JNDs were undefined for the 100 Hz standard for all tested contacts. Note that participants could perceive the stimuli even when the JND was undefined, but they could not discriminate between them.

#### Effect of pulse width on frequency discrimination

Second, we examined the degree to which PF could be discriminated independently of changes in perceived magnitude. Trials with equal-PW pairs were interleaved with trials in which the PW of the comparison was different from that of the standard stimulus. To the extent that participants relied on differences in perceived magnitude to make their frequency judgments, PW would systematically bias participants' frequency judgments, thereby causing lateral shifts in psychometric functions. Changes in PW may also lead to changes in the clarity or vividness of the evoked percepts, thereby resulting in changes in the discriminability of PF.

**Figure 4.**
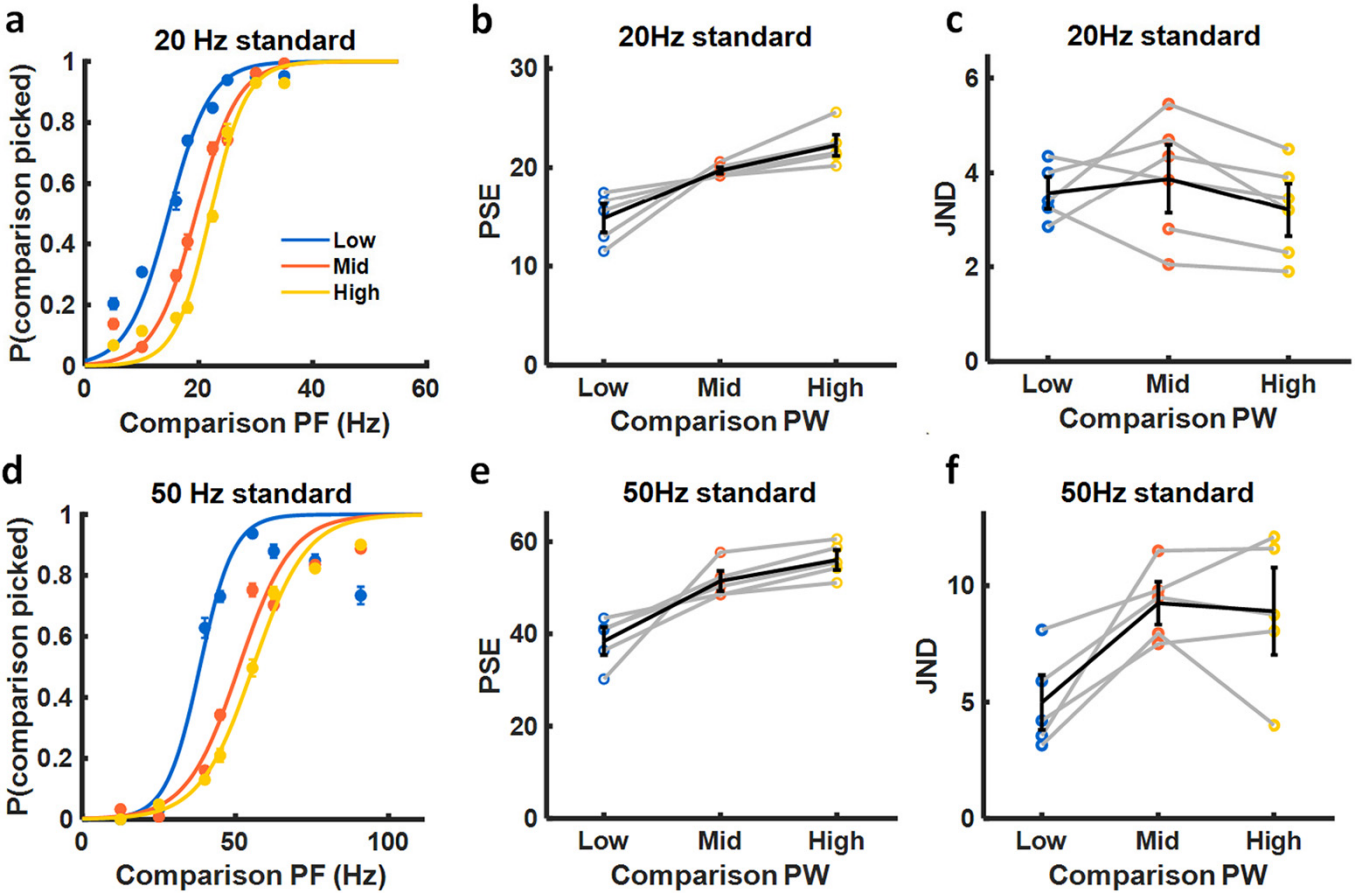
PF discrimination performance with variable PW. ***a***, Performance with the 20 Hz standard and three comparison PWs, averaged across electrode contacts (*n* = 6). ***b***, PSE versus PW for the 20 Hz standard (*n* = 17). Gray lines denote different contacts, black line denotes the mean across contacts and participants. PSE increases with PW, revealing a PW-dependent bias in PF discrimination performance. Participants exhibited a tendency to perceive higher PWs as being lower in PF. ***c***, JND versus PW for the 20 Hz standard (*n* = 17). There was no significant effect of PW on JND with the 20 Hz standard. ***d***, Performance with the 50 Hz standard and three comparison PWs, averaged across electrode contacts (*n* = 5). ***e***, PSE versus PW for the 50 Hz standard (*n* = 15). PSE was significantly higher at higher PWs. ***f***, JND versus PW for the 50 Hz standard (*n* = 15). JNDs tended to be higher at higher PWs.

We observed systematic leftward shifts in the psychometric functions when the comparison PW was lower than the standard PW and systematic rightward shifts when the comparison PW was higher than the standard PW (Fig. 4*a*,*b*,*d*,*e*). In other words, pulse trains with lower PW tended to be perceived as higher in frequency across PFs. This bias was quantified by computing the PSE—the PF at which participants were equally likely to select the standard as they were the comparison stimulus. The PSE increased as the comparison PW increased for both the 20 and 50 Hz standards (Fig. 4*b*,*e*; repeated-measures ANOVA: *F*_(2,16)_ = 17.34, *p* < 0.001; *F*_(2,14)_ = 17.46, *p* = 0.0012, respectively). In summary, the participants exhibited a slight tendency to select the more intense stimulus as being lower in frequency, consistent with previous findings that the pitch of a vibrotactile stimulus decreases as the stimulus amplitude increases over this range of frequencies (Roy and Hollins, 1998; Prsa et al., 2021). Note that this tendency was also observed in the frequency estimation ratings but was too weak to achieve statistical significance.

Next, we investigated the degree to which the comparison PW affected participants' sensitivity to changes in PF by examining the effect of PW on the JND. For the 20 Hz standard, we found no systematic effect of PW on JND (Fig. 4*c*; repeated-measures ANOVA: *F*_(2,16)_ = 1.35, *p* = 0.3082). For the 50 Hz standard, on the other hand, JNDs increased as PW increased (Fig. 4*f*; *F*_(2,14)_ = 9.58, *p* = 0.0075). In other words, participants became somewhat less sensitive to changes in PF at higher PWs, but only for the higher PF standard.

### Subjective reports of sensory quality depend on stimulation frequency

Given that PF reliably influenced perceived quality along a single frequency continuum, we then investigated how PF shapes subjective quality more broadly. To this end, participants indicated which subset of a list of 30 qualitative descriptors pertained to each stimulus. Participants were encouraged to select as many words as necessary to describe the electrically evoked sensation.

#### Qualitative reports

On average, participants selected 6.5 + 2.6 words (mean ± SEM) to describe each sensation. The total number of words chosen to describe a stimulus varied across participants, ranging from 5 to 22.

**Figure 5.**
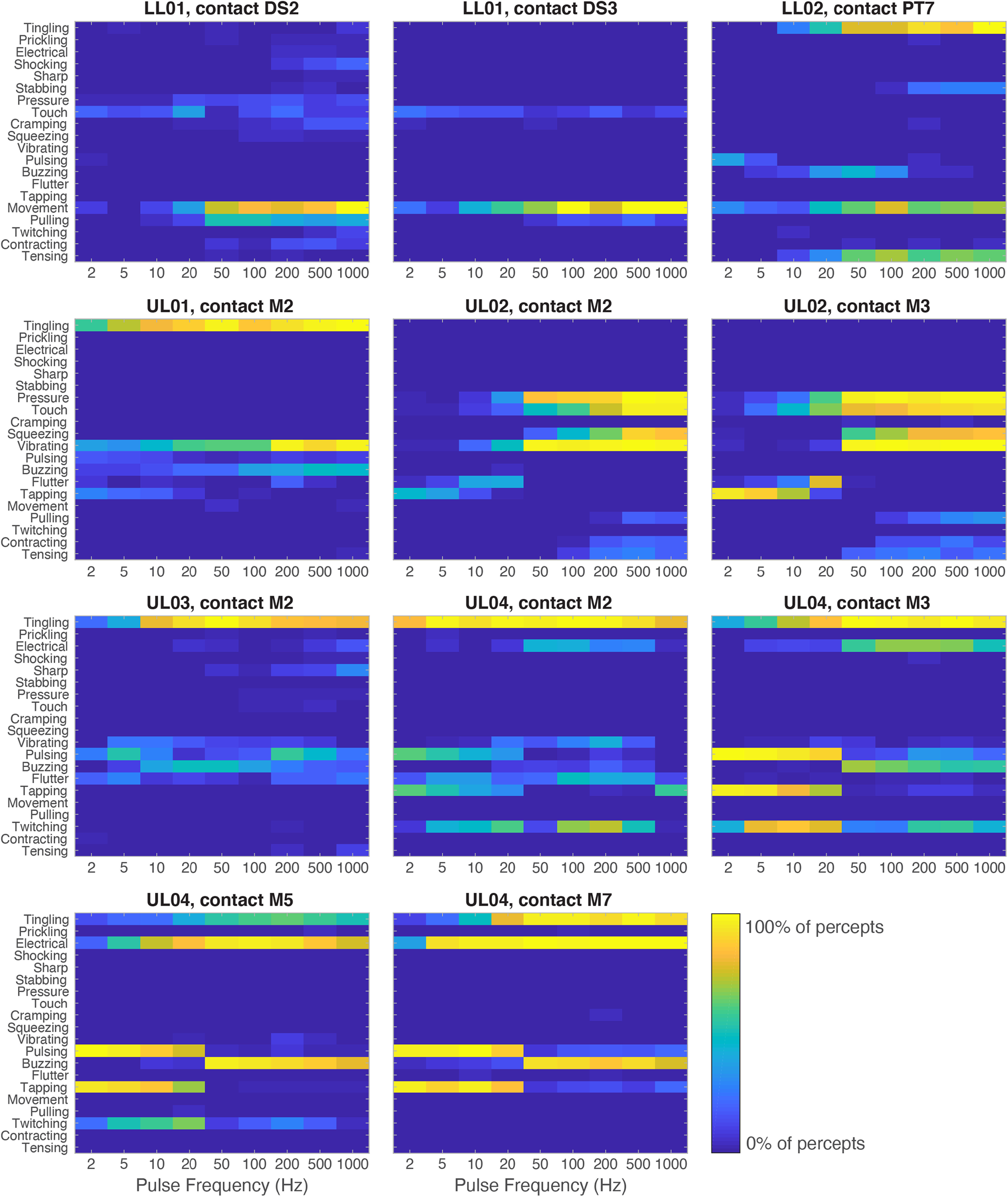
Quality descriptors reported for electrically evoked percepts at each PF, averaged across PWs. Each heatmap corresponds to a participant and contact (*n* = 11), and each row corresponds to a descriptor. Electrode contacts are labeled as nerve and contact number (DS = distal sciatic, PT = proximal tibial, M= median). Color denotes the proportion of times a descriptor was selected at each PF. Participants used different words to describe their sensory experiences. However, descriptors were similar across contacts for each participant.

Examination of the participants' word selections revealed that stimulation PF influenced the descriptors chosen to characterize the stimuli. That is, different sets of words were selected to describe low-frequency stimuli than high-frequency stimuli (Fig. 5, Table 1), as has been found with vibrotactile stimulation (Talbot et al., 1968) and intracortical microstimulation (Hughes et al., 2021). For example, descriptors related to periodic sensations, such as “tapping,” “pulsing,” “twitching,” and “flutter,” were commonly selected for low-PF stimuli, whereas continuous sensations, such as “tingling,” “buzzing,” “electrical,” “pressure,” and “touch, were reported for high-PF stimuli (Table 1). In addition, the total number of reported descriptors increased as a function of stimulation PF (linear regression, *p* < 0.001).

**Table 1.** The selection rate for perceptual descriptors differed for low-frequency and high-frequency stimulation conditions

Low frequency (<50 Hz)		High frequency (≥50 Hz)	
Descriptor	Selection rate	Descriptor	Selection rate
Tapping	36%	Tingling	49%
Pulsing	34%	Buzzing	28%
Tingling	29%	Electrical	26%
Twitching	15%	Vibrating	22%
Electrical	14%	Pressure	13%
Flutter	7%	Touch	12%
Vibrating	6%	Movement	9%

Selection rates are given as the percentage of total trials that each descriptor word was reported across all participants, contacts, and PWs. The top seven most frequently reported words for each frequency regime are shown. Note that since multiple words can be selected for a single trial, the selection rates will not sum to 100%.

Participants used similar descriptors across electrode contacts, as evidenced by highly correlated descriptor selection within participants (*R*^2^ = 0.66 ± 0.08, mean ± SEM). The consistency in reports across electrode contacts indicates that quality is largely independent of which region of the nerve is stimulated. However, the descriptors ascribed to individual stimuli varied widely from participant to participant (Fig. 5), yielding weak correlations in descriptor selections across participants (*R*^2^ = 0.14 ± 0.04). These variations likely reflect the participants' idiosyncratic descriptor preferences, since some words, such as tapping and pulsing, may be construed as synonymous.

#### A robust representation of sensory quality

**Figure 6.**
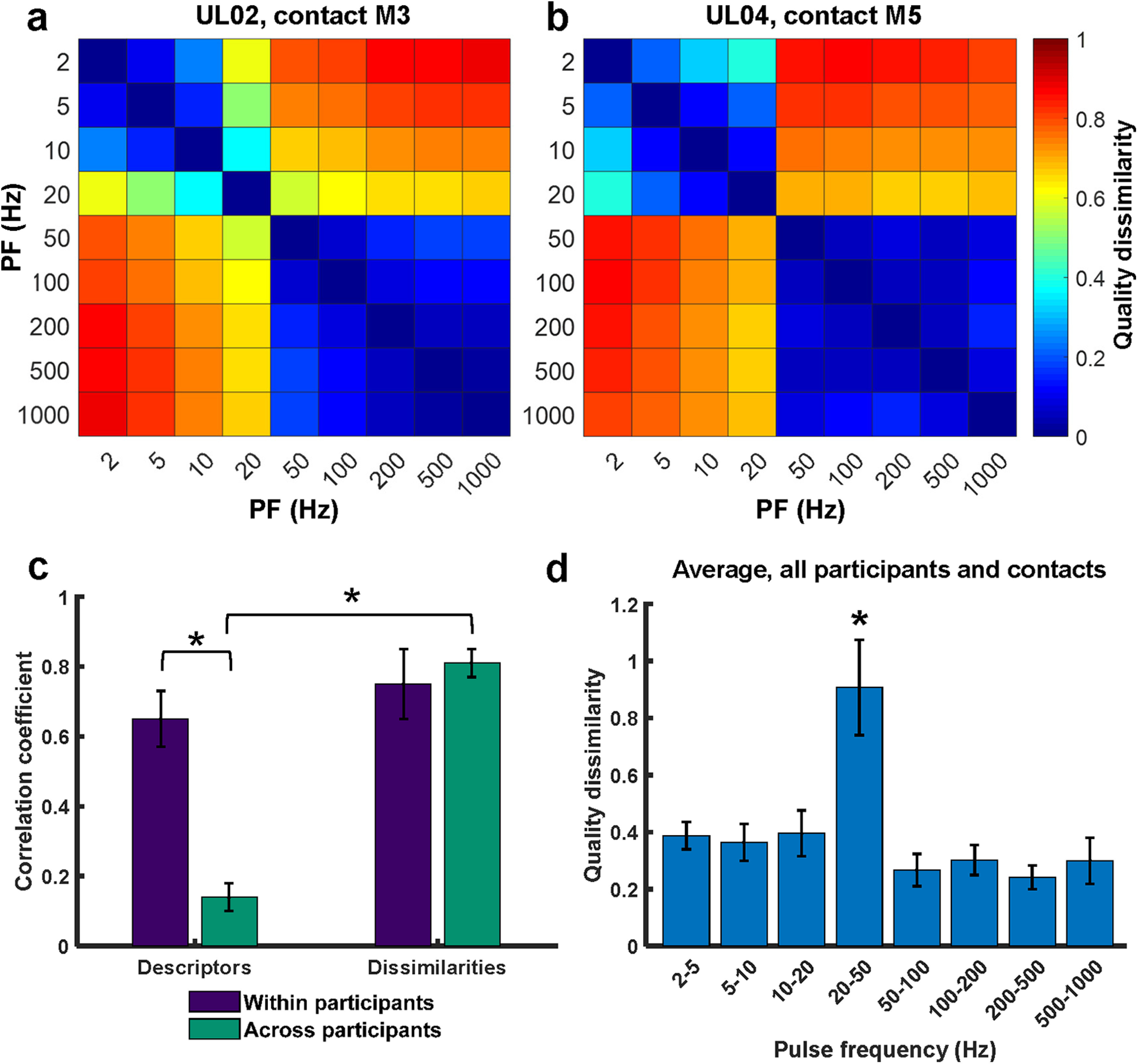
Perceived dissimilarity estimated from differences in descriptor usage. ***a***, Perceived dissimilarity of stimuli varying in PF for contact M3 of participant UL02. ***b***, Perceived dissimilarity of stimuli varying in PF for contact M5 of participant UL04. ***c***, Within-participant (*n* = 8, purple) and across-participant (*n* = 15, teal) pairwise correlations for two metrics of quality. “Descriptors” denotes the words selected by the participant for each stimulus, and “Dissimilarities” denotes the Euclidean distance between pairs of stimulation conditions. Error bars denote the SEM. Asterisks denote statistically significant differences with *p* < 0.001. ***d***, Perceived dissimilarity between successive PF conditions, averaged across PWs (*n* = 33). Error bars indicate the SEM. The asterisk denotes significance at *p* < 0.001.

To overcome participant-specific descriptor choice and assess the effect of PF on quality across participants, we computed differences in descriptor selection between each pair of stimuli. To this end, we first represented quality as a 30-element vector, where each element corresponded to the proportion of trials in which a given descriptor was selected. We then ran a PCA on the vectors of individual participants to consolidate redundant or highly correlated descriptors. Finally, we computed the Euclidean distance between each pair of stimulation conditions (PW and PF combinations) in this reduced dimensional space. These distances represent the perceived dissimilarity between pairs of stimuli, estimated from the differences in descriptor selection (Fig. 6*a*,*b*).

We found that the resulting dissimilarity matrices were highly consistent across participants and electrode contacts (Fig. 6*a*,*b*). Indeed, dissimilarity matrices were significantly more similar across participants (*R*^2^ = 0.81 ± 0.04) than were the descriptor selections from which they were derived (*R*^2^ = 0.14 ± 0.04; one-way ANOVA: *F*_(3,42)_ = 35.80, *p* < 0.001; Fig. 6*c*, teal bars). The consistency in dissimilarity matrices across participants suggests that the relationship between sensation quality and PF is similar across participants, despite idiosyncratic descriptor usage.

#### Impact of PF on overall quality

Having established a robust representation of multidimensional quality based on dissimilarity, we then assessed the degree to which changes in PF led to changes in the quality of the evoked percept. We found a sharp transition in sensation quality between 20 and 50 Hz for all tested contacts. In fact, the dissimilarity between 20 and 50 Hz stimuli was significantly greater than the dissimilarity between any other pair of adjacent frequencies (ANOVA: *F*_(7,197)_ = 16.14, *p* < 0.001; Fig. 6*d*). In other words, the perceptual quality was consistent across PFs ranging from 2 to 20 Hz and across PFs from 50 to 1000 Hz, but these two subsets of stimuli felt very different from one another. There was no systematic effect of stimulation PW on the dissimilarities between successive PFs (ANOVA: *F*_(2,197)_ = 1.22, *p* = 0.3).

**Figure 7.**
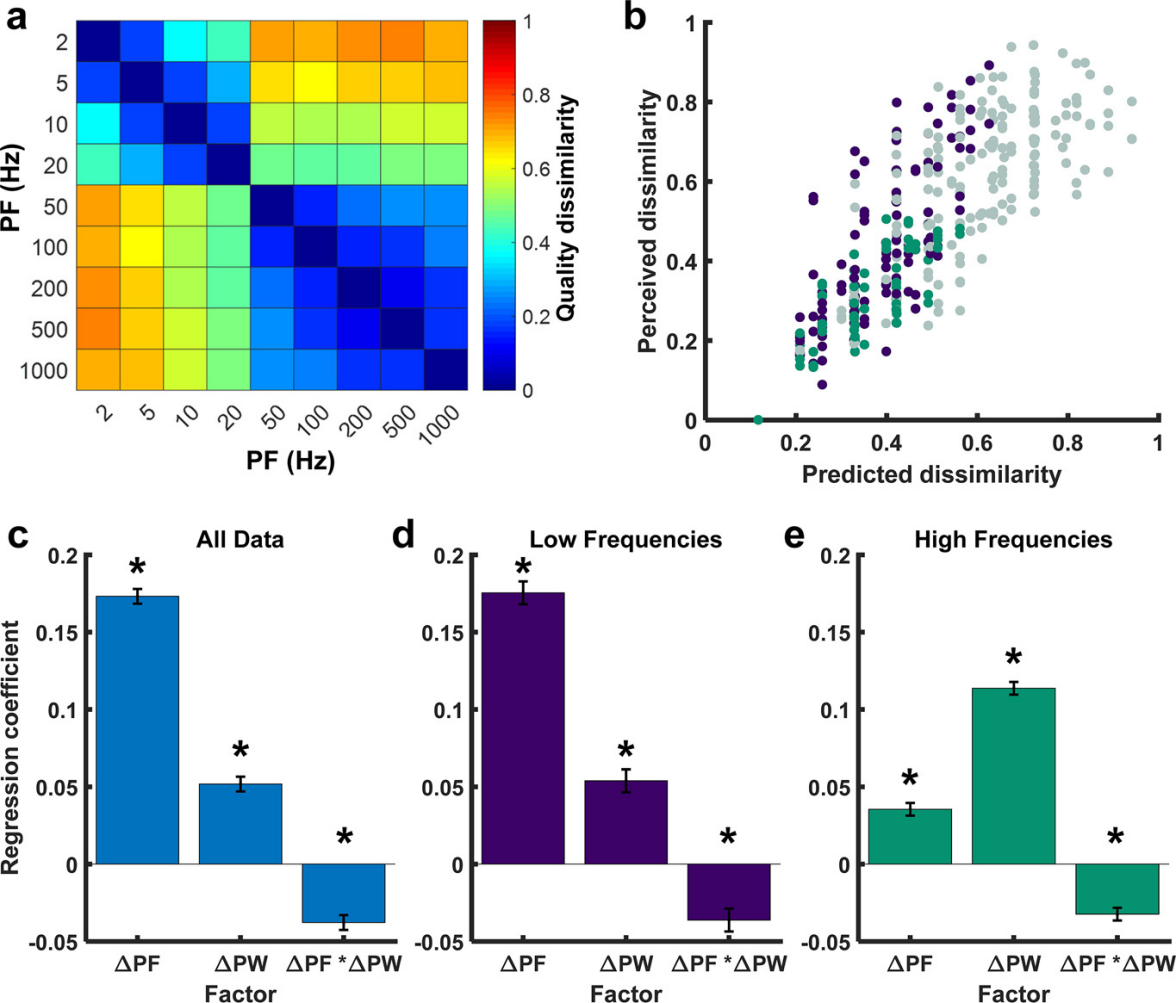
Contribution of stimulation parameters to sensation quality. ***a***, Dissimilarity matrix averaged across participants and contacts (*n* = 11). ***b***, Measured dissimilarity versus prediction from a linear combination of ΔPF, ΔPW, and their interaction. Purple points represent dissimilarities between stimuli at low frequencies (<50 Hz), green points represent dissimilarities between stimuli at high frequencies (>50 Hz), and gray points represent dissimilarities between high-frequency and low-frequency stimuli. ***c***, Standardized regression coefficients for the model with all data included. ***d***, Regression coefficients for the model with only low-frequency data included (<50 Hz). ***e***, Regression coefficients for the model with only high-frequency data included (>50 Hz). In ***c–e***, Error bars are SE of the estimates. Asterisks denote significant contribution to the model at *p* < 0.001.

We then determined the extent to which differences in PF and PW resulted in differences in the evoked sensation. To quantify the impact of PF and PW on quality, we averaged the dissimilarity matrices across contacts and participants (Fig. 7*a*) and regressed the resulting matrix on differences in PF or PW (ΔPF, ΔPW). The regression model that included both ΔPF and ΔPW yielded accurate predictions of dissimilarity (*R*^2^ = 0.67, *F*_(3,725)_ = 499, *p* < 0.001; Fig. 7*b*). While both ΔPF and ΔPW had significant impacts on dissimilarity, the contribution of ΔPF was much higher than that of ΔPW (standardized coefficients: β_ΔPF_ = 0.17, *p* < 0.001; β_ΔPW_ = 0.05, *p* < 0.001; Fig. 7*c*). The interaction between ΔPF and ΔPW was significant but weak (β_ΔPF*ΔPW_ = −0.04, *p* < 0.001). We performed the same analyses on individual participants' data and obtained the same results, confirming that these results were not an artifact of pooling.

Given the sharp transition in perceived quality ∼50 Hz, we fit separate regression models for the dissimilarity data at <50 Hz and >50 Hz. At low PFs, the regression was nearly identical to that obtained using the full dataset (*R*^2^ = 0.74, *F*_(3,221)_ = 214, *p* < 0.001; β_ΔPF_ = 0.18, *p* < 0.001; β_ΔPW_ = 0.05, *p* < 0.001; β_ΔPF*ΔPW_ = −0.04, *p* < 0.001; Fig. 7*d*). In contrast, at high PFs, the effect of PW dominated that of PF (*R*^2^ = 0.74, *F*_(3,140)_ = 302, *p* < 0.001; β_ΔPF_ = 0.04, *p* < 0.001; β_ΔPW_ = 0.11, *p* < 0.001; β_ΔPF*ΔPW_ = −0.03, *p* < 0.001; Fig. 7*e*).

#### Relationship between perceived frequency and dissimilarity

**Figure 8.**
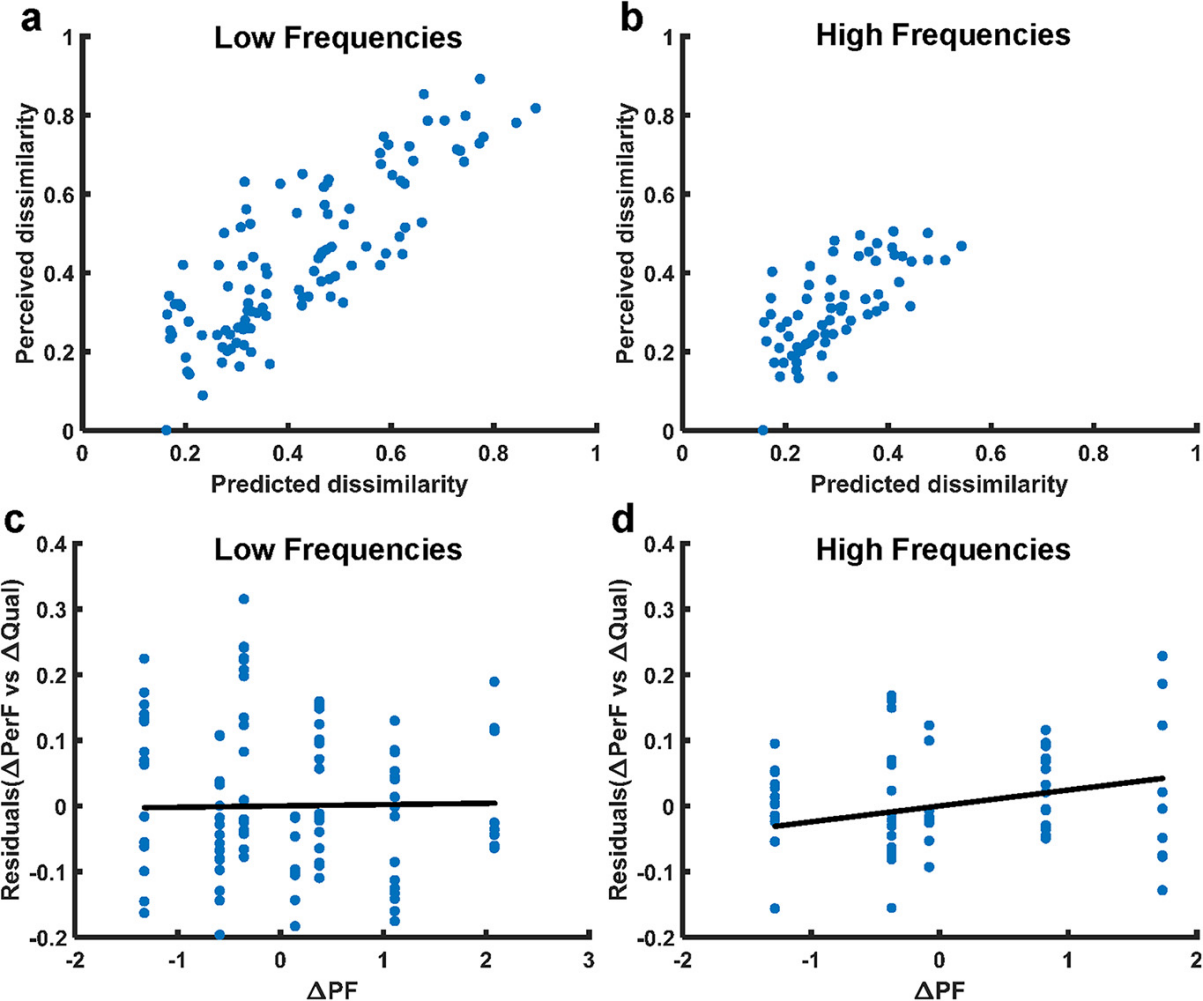
Effect of PF on perceived dissimilarity after removing the influence of perceived frequency. ***a***, ***b***, A univariate regression predicting quality dissimilarity (ΔQual) based on perceived frequency (ΔPerF) was performed for low-frequency (***a***) and high-frequency (***b***) stimuli. Perceived dissimilarity is plotted versus the prediction from the regression. ***c***, ***d***, Residuals of the regression (shown in ***a*** and ***b***) versus (*z*-scored) ΔPF. Solid black lines denote the regression of the residuals on to ΔPF. ***c***, At low frequencies, the influence of ΔPF on dissimilarity was not significant after accounting for perceived frequency (*p* = 0.8). ***d***, At high frequencies, ΔPF significantly impacted dissimilarity, even after accounting for perceived frequency (*p* = 0.001), but the effect was modest.

Finally, we determined whether the effect of PF on dissimilarity could be explained solely based on its effect on perceived frequency. That is, we examined whether PF modulates perceptual quality along a single continuum. To this end, we first regressed dissimilarity on the perceived frequency ratings obtained in the first experiment, then assessed whether the residuals covaried with ΔPF. In the low-frequency regime, we found that differences in perceived frequency predicted dissimilarity ratings well (*R*^2^ = 0.71, *F*_(1,223)_ = 545, *p* < 0.001; Fig. 8*a*) and that ΔPF was not significantly predictive of the residuals (*R*^2^ = −0.004, *F*_(1,223)_ = 0.07, *p* = 0.80; Fig. 8*c*). Thus, the effect of PF on quality is confined to a single continuum in the low-frequency regime.

In the high-frequency regime, we similarly found that perceived frequency was a significant predictor of dissimilarity, as it was in the low-frequency regime (*R*^2^ = 0.53, *F*_(1,142)_ = 164; *p* < 0.001; Fig. 8*b*). However, ΔPF continued to have a weak but significant effect on dissimilarity ratings after regressing out the effect of perceived frequency (*R*^2^ = 0.07, *F*_(1,142)_ = 11, *p* = 0.001; Fig. 8*d*). Thus, in the high-frequency regime, the effect of PF on quality dissimilarity cannot be explained purely on the basis of perceived frequency, consistent with the finding that perceived frequency is not dependent on PF at those frequencies (Fig. 2). Note, however, that the overall impact of PF on quality was weak in the high-frequency range (Fig. 7*e*), so the magnitude of these effects was small.

## Discussion

### Stimulation frequency shapes sensory quality <50 Hz

We find that the temporal pattering in the neuronal response, which in this case was periodic and defined by PF, shapes the perceived quality of electrically evoked tactile percepts both along a single continuum—perceived frequency—and across a multidimensional space defined by perceptual descriptors. The effect of PF on quality was most pronounced at low PFs, below ∼50 Hz. In this frequency range, increases in PF led to increases in perceived frequency and systematic changes in verbal reports of sensory quality. In addition, we found that the impact of PF on verbally reported quality could be accounted for by variations in perceived frequency, demonstrating that PF modulates quality along a single continuum. The fact that PW did not have a significant effect on perceived frequency and had a marginal effect on overall quality in this frequency range indicates that the effect of PF on quality does not simply reflect a change in perceived magnitude.

Together, these results show that temporal patterning in the aggregate afferent response can modulate the quality of tactile percepts at low frequencies, despite the fact that this temporal patterning is distributed across afferents of all classes. Thus, the differential recruitment of afferent classes based on their frequency sensitivity profiles is not the only factor that shapes quality perception, though it likely plays an important role (Ochoa and Torebjörk, 1983; Saal and Bensmaia, 2014; Birznieks et al., 2019).

### Changes in PF are indiscriminable at high frequencies

Changing the frequency of vibrations delivered to the skin results in a change in the sensory experience along a continuum described as “vibrotactile pitch,” underscoring its similarity to the sensory consequences of changes in the frequency of an acoustic tone (Roy and Hollins, 1998; Yau et al., 2009). Human observers can reliably discriminate a change in vibrotactile frequency of ∼20%, but sensitivity to changes in frequency decreases at higher vibration frequencies (Goff, 1967; Mountcastle et al., 1969, 1990; LaMotte and Mountcastle, 1975). Changes in vibrotactile frequency also produce changes in perceived magnitude (Verrillo et al., 1969). However, human participants can discriminate vibratory frequency in the presence of concomitant and independent changes in vibratory amplitude (Harvey et al., 2013), demonstrating that vibrotactile frequency also shapes the quality of the percept.

The ability to perceive the frequency of a vibration is thought to be mediated by a timing code in the nerve (Birznieks and Vickery, 2017; Birznieks et al., 2019). Sinusoidal vibrations delivered to the skin elicit periodic, phase-locked spiking responses in tactile nerve fibers, and the frequency of these responses matches the frequency of stimulation (Talbot et al., 1968; Mackevicius et al., 2012). When this temporal patterning is absent, for example at low stimulus amplitudes, the ability to discriminate frequency is abolished (Talbot et al., 1968).

Pulsed electrical stimulation of the nerve also produces a highly periodic response in the activated tactile nerve fibers (Formento et al., 2020), and this frequency-dependent temporal patterning likely mediates the ability to discriminate the perceived frequency of electrical pulse trains. In fact, sensitivity to changes in the frequency of electrical stimulation is similar to its vibrotactile counterpart, both yielding Weber fractions of ∼0.2. We also demonstrate that stimulation PF has a larger effect on the quality of an electrically evoked sensation than it does on its magnitude: the Weber fraction for PF along the perceived magnitude continuum (0.3; Graczyk et al., 2016) is higher than that along the perceived frequency continuum.

In contrast to vibrotactile frequency discrimination, which can be achieved over a wide range of frequencies up to 400 Hz and beyond (Goff, 1967; Harvey et al., 2013), we show that frequency discrimination of peripheral nerve stimulation is only possible at frequencies below ∼50 Hz. Above this point, frequency discrimination falls to near chance. This is surprising in light of the fact that nonhuman primates can distinguish the PF of intracortical microstimulation up to ∼200 Hz, though sensitivity to changes in PF decreases at high frequencies (Callier et al., 2020). While prior studies of electrocutaneous stimulation reported discriminability of pulse trains up to 100 Hz (Anani et al., 1977; Szeto et al., 1979; Ng et al., 2020, 2021), the psychophysical tasks in these prior studies were not designed to eliminate intensity cues; so, results therefrom are difficult to compare with those reported here. Indeed, intensity discrimination of direct nerve stimulation has also been observed up to 100 Hz (Graczyk et al., 2016). Thus, while changes in PF can be discerned at >50 Hz, this ability may not rely on perceived frequency.

In addition, while our results showed that pulse trains with higher PW tended to be judged as lower in frequency, prior studies of intracortical microstimulation in nonhuman primates found that stimuli with higher PA tended to be reported as being higher in frequency (Callier et al., 2020). Assuming that PW and PA have similar neural and sensory correlates, the impact of intensity on frequency discrimination is thus different for the nerve and cortex. The basis for these differences is unclear, but the neural consequences of stimulation differ between nerve—a bundle of independent channels—and cortex—a densely interconnected circuit.

### Perceived dissimilarity is a robust way to quantify quality

Quality is difficult to quantify because this aspect of a sensory experience occupies a high dimensional space and measuring it relies on verbal reports, which are often highly idiosyncratic. In this study, the specific words chosen to describe the qualitative sensory experiences varied widely across participants. To mitigate this variability in descriptor selection, we developed an approach to represent quality based on *differences* in the descriptors that participants ascribed to the sensations. We found that this representation of quality was very similar across participants, suggesting that tactile sensations evoked from neural stimulation may be approximately equivalent across individuals despite idiosyncratic descriptor usage. We propose that our approach could be used in future studies of quality perception to improve comparisons across participants when the perceptual space is not confined to one or a few experimenter-defined dimensions.

### Quality abruptly changes at 50 Hz

As discussed above, the ability to discriminate PF was abolished beyond ∼50 Hz, and the influence of PF on quality was far more pronounced at low PFs. In contrast, PW had a greater impact on quality at high PFs than did PF. Furthermore, multidimensional quality also shifted abruptly at ∼50 Hz.

There are several possible explanations for the transition between the low-frequency and high-frequency regimes. One possibility is that the tactile nerve fibers cannot, as a population, phase lock to stimulation pulse trains beyond ∼50 Hz. Indeed, the temporal patterning in the neural response likely drives the effect of stimulation PF on sensory quality. At high frequencies, stimulation pulses may encroach on the refractory period resulting from the neural response to the previous pulse. This phenomenon might then blur the temporal patterning in the population response. However, given that the refractory period is typically <5 ms (Ochoa and Torebjörk, 1983) and that pulses are separated by 20 ms at 50 Hz, refractoriness is unlikely to play a critical role in the observed transition in quality.

Another possibility is that the spikes evoked in the afferent population by each pulse get desynchronized downstream because of conduction delays, thereby blurring the temporal patterning at the population level. Indeed, neural conduction velocities vary across Aβ nerve fibers that mediate touch because of the natural variance in fiber diameters (Johansson and Vallbo, 1983). In an adult male, the resulting propagation delays for signals to travel from the fingertip to spinal cord range from 11 to 22 ms (Hursh, 1939). However, this jitter is not observed in the vibrotactile responses of neurons in the somatosensory cortex (Mountcastle et al., 1969; Harvey et al., 2013), which exhibit a high degree of phase locking, suggesting that some compensatory mechanism might eliminate this endogenous jitter along the way to the brain. The synchronized spikes delivered through a nerve cuff might then become desynchronized via this compensatory mechanism. Given that the intrinsic delay correction would span ∼10 ms (to correct for delays ranging from 11 to 22 ms) and that the cuff electrode is positioned approximately halfway between the fingertip and spinal cord, the imposed jitter would span ∼5 ms. We might then expect frequency discrimination to break down around 200 Hz. Thus, this conduction delay-mediated desynchronization mechanism is also unlikely to be solely responsible for the transition at 50 Hz.

A more likely possibility is that the unnatural activation of the afferent submodalities by peripheral nerve stimulation obscures the temporal pattern imposed by stimulation at high PFs. Electrical stimulation recruits Aβ fibers based on their fiber diameter and nodal voltages (Richardson et al., 2000; McIntyre et al., 2002), regardless of their submodality class. In natural touch, the various submodalities of tactile fibers differ in the frequency profiles of their sensitivity to skin vibrations: slowly adapting type 1 (SA1) fibers respond preferentially to low-frequency vibrations; Pacinian corpuscle (PC)-associated fibers respond preferentially to high-frequency vibrations; and rapidly adapting (RA) fibers exhibit intermediate frequency preferences (Talbot et al., 1968; Freeman and Johnson, 1982; McGlone and Reilly, 2010). Given the pulse amplitudes used in this study, tens or hundreds of nerve fibers are likely recruited (Graczyk et al., 2016). A high-PF pulse train will thus activate SA1 fibers, and perhaps RA fibers, to a much greater extent than would a high-frequency vibration delivered to the skin. This large, unnatural signal may obscure the critical signal carried by appropriately responding afferents, especially PC fibers. This phenomenon would be further magnified by the fact that RA1 and SA1 fibers far outnumber PC fibers in the periphery (a ratio of 1:1.6:0.6 for SA1:RA1:PC fibers; Johansson and Vallbo, 1979).

The hypothesis that overactivation of SA1 and RA fibers could be implicated in poor frequency perception at high PFs is supported by previous studies of intraneural microstimulation: while increases in the PF delivered to individual RA afferents induce increases in perceived frequency at low PFs, further increases only lead to increased intensity (Torebjörk et al., 1987). Stimulation of individual SA1 fibers leads to sustained percepts at all but the lowest PFs (Torebjörk et al., 1987). The submodality overactivation hypothesis is also bolstered by the previous finding that the impact of peripheral afferent signals on cortical neural activity is highly dependent on the submodality of the peripheral afferents: PC signals in the periphery drive the temporal patterning of neural responses in cortex, whereas RA and SA1 signals instead drive the strength of the response in cortex (Saal et al., 2015). Thus, the unnatural strength of the SA1 signal (and perhaps the RA signal) evoked via peripheral nerve stimulation may muddle the crucial frequency signal transmitted by PCs to the cortex. This phenomenon would be exacerbated at high PFs, at which SA1 fibers are typically relatively quiescent, consistent with our results. A similar phenomenon, in which RA signals interfere with SA1 signals about local geometric features, has been previously documented (Bensmaïa et al., 2006).

### Dependence of quality on PF is consistent across fascicles

We did not observe any electrode-specific differences in the impact of PF on perceived quality, and prior studies similarly found no electrode-specific differences in intensity perception (Graczyk et al., 2016). In contrast, the perceptual correlates of changes in the PF of intracortical microstimulation have been shown to vary across electrodes in experiments with human subjects (Hughes et al., 2021) and nonhuman primates (Callier et al., 2020). These results have been interpreted as reflecting differences in the functions of the stimulated cortical circuits (Sur et al., 1981, 1984; Pei et al., 2009; Saal and Bensmaia, 2014). Conversely, the fascicles of the peripheral nerve display only minimal submodality-specific organization (Ekedahl et al., 1997; Wu et al., 1999; Hallin and Wu, 2001), so the submodality composition of the nerve fibers activated through electrical stimulation is likely to be consistent across electrode contacts.

### Stimulation frequency can be used to sculpt artificial touch for bionic limbs

A major challenge facing recent efforts to restore the sense of touch through neural stimulation is to evoke sensations endowed with an appropriate quality. The goal is for contact with a textured surface through a bionic hand to lead to a perceived sensation of the appropriate texture and contact with an edge to lead to the sensation of an edge. In the intact sensory system, the evoked percept and the spatiotemporal pattern of activation elicited in the nerve when interacting with an object depend on the dynamics of contact and the properties of the object. While electrical stimulation of the nerves can reproduce the temporal aspects of this natural neural activity by patterning the pulses, it cannot mimic the complex spatiotemporal patterns because of limitations in the ability to selectively activate individual nerve fibers. Nonetheless, mimicking natural patterns of activation with biomimetic stimulation enables the discrimination of textural features (Oddo et al., 2016), confers greater speed to certain sensory discrimination tasks performed with a prosthesis (George et al., 2019), and improves performance on tasks involving object manipulation (Valle et al., 2018). In contrast, the degree to which biomimetic stimulation modulates the quality and naturalness of the sensation is inconsistent (Graczyk, 2018; Valle et al., 2018; George et al., 2019). Our results demonstrate that modulating stimulation frequency reliably and systematically modulates sensation quality. This phenomenon can be leveraged to convey information about object and substrate interactions more intuitively for upper and lower extremity neuroprostheses.

## References

[B1] Adrian ED (1919) The response of human sensory nerves to currents of short duration. J Physiol 53:70–85. 10.1113/jphysiol.1919.sp001860 16993441 PMC1405594

[B2] Anani AB, Ikeda K, Korner LM, Körner LM (1977) Human ability to discriminate various parameters in afferent electrical nerve stimulation with particular reference to prostheses sensory feedback. Med Biol Eng Comput 15:363–373. 10.1007/BF02457988 197328

[B3] Bensmaïa SJ, Craig JC, Johnson KO (2006) Temporal factors in tactile spatial acuity: evidence for RA interference in fine spatial processing. J Neurophysiol 95:1783–1791. 10.1152/jn.00878.2005 16236778 PMC1839044

[B4] Bergmann Tiest WM, Kappers AML (2006) Analysis of haptic perception of materials by multidimensional scaling and physical measurements of roughness and compressibility. Acta Psychol (Amst) 121:1–20. 10.1016/j.actpsy.2005.04.005 16055070

[B5] Birznieks I, Vickery RM (2017) Spike timing matters in novel neuronal code involved in vibrotactile frequency perception. Curr Biol 27:1485–1490.e2. 10.1016/j.cub.2017.04.011 28479322

[B6] Birznieks I, McIntyre S, Nilsson HM, Nagi SS, Macefield VG, Mahns DA, Vickery RM (2019) Tactile sensory channels over-ruled by frequency decoding system that utilizes spike pattern regardless of receptor type. eLife 8:e46510. 10.7554/eLife.46510 31383258 PMC6684274

[B7] Callier T, Brantly NW, Caravelli A, Bensmaia SJ (2020) The frequency of cortical microstimulation shapes artificial touch. Proc Natl Acad Sci U S A 117:1191–1200. 10.1073/pnas.1916453117 31879342 PMC6969512

[B8] Davis TS, Wark HAC, Hutchinson DT, Warren DJ, O'Neill K, Scheinblum T, Clark GA, Normann RA, Greger B (2016) Restoring motor control and sensory feedback in people with upper extremity amputations using arrays of 96 microelectrodes implanted in the median and ulnar nerves. J Neural Eng 13:036001. 10.1088/1741-2560/13/3/036001 27001946

[B9] Dhillon GS, Horch KW (2005) Direct neural sensory feedback and control of a prosthetic arm. IEEE Trans Neural Syst Rehabil Eng 13:468–472. 10.1109/TNSRE.2005.856072 16425828

[B10] Ekedahl R, Frank O, Hallin RG (1997) Peripheral afferents with common function cluster in the median nerve and somatotopically innervate the human palm. Brain Res Bull 42:367–376. 10.1016/s0361-9230(96)00324-3 9092878

[B11] Formento E, D'Anna E, Gribi S, Lacour SP, Micera S (2020) A biomimetic electrical stimulation strategy to induce asynchronous stochastic neural activity. J Neural Eng 17:046019. 10.1088/1741-2552/aba4fc 32650319

[B12] Freeberg MJ, Stone MA, Triolo RJ, Tyler DJ (2017) The design of and chronic tissue response to a composite nerve electrode with patterned stiffness. J Neural Eng 14:036022. 10.1088/1741-2552/aa6632 28287078 PMC5713483

[B13] Freeman AW, Johnson KO (1982) A model accounting for effects of vibratory amplitude on responses of cutaneous mechanoreceptors in macaque monkey. J Physiol 323:43–64. 10.1113/jphysiol.1982.sp014060 7097579 PMC1250344

[B14] Geng B, Yoshida K, Petrini L, Jensen W (2012) Evaluation of sensation evoked by electrocutaneous stimulation on forearm in nondisabled subjects. J Rehabil Res Dev 49:297–308. 10.1682/JRRD.2010.09.0187 22773530

[B15] George JA, Kluger DT, Davis TS, Wendelken SM, Okorokova EV, He Q, Duncan CC, Hutchinson DT, Thumser ZC, Beckler DT, Marasco PD, Bensmaia SJ, Clark GA (2019) Biomimetic sensory feedback through peripheral nerve stimulation improves dexterous use of a bionic hand. Sci Robot 4:eaax2352. 10.1126/scirobotics.aax2352 33137773

[B16] Goff GD (1967) Differential discrimination of frequency of cutaneous mechanical vibration. J Exp Psychol 74:294–299. 10.1037/h0024561 6048472

[B17] Goodman JM, Bensmaia SJ (2018) The neural basis of haptic perception. In: Stevens' handbook of. experimental psychology and cognitive neuroscience, Vol 2 (Wixted JT, ed), pp 1–39. Newark, NJ: Wiley.

[B18] Graczyk EL (2018) Natural perceptual characteristics and psychosocial impacts of touch evoked by peripheral nerve stimulation. PhD thesis, Case Western Reserve University.

[B19] Graczyk EL, Schiefer MA, Saal HP, Delhaye BP, Bensmaia SJ, Tyler DJ (2016) The neural basis of perceived intensity in natural and artificial touch. Sci Transl Med 8:362ra142. 10.1126/scitranslmed.aaf5187 PMC5713478 27797958

[B20] Graczyk EL, Delhaye BP, Schiefer MA, Bensmaia SJ, Tyler DJ (2018) Sensory adaptation to electrical stimulation of the somatosensory nerves. J Neural Eng 15:046002. 10.1088/1741-2552/aab790 29551756 PMC6034502

[B21] Guest S, Dessirier JM, Mehrabyan A, McGlone F, Essick G, Gescheider G, Fontana A, Xiong R, Ackerley R, Blot K (2011) The development and validation of sensory and emotional scales of touch perception. Atten Percept Psychophys 73:531–550. 10.3758/s13414-010-0037-y 21264727

[B22] Hallin R, Wu G (2001) Fitting pieces in the peripheral nerve puzzle. Exp Neurol 172:482–492. 10.1006/exnr.2001.7813 11716573

[B23] Harvey MA, Saal HP, Dammann JF, Bensmaia SJ (2013) Multiplexing stimulus information through rate and temporal codes in primate somatosensory cortex. PLoS Biol 11:e1001558. 10.1371/journal.pbio.1001558 23667327 PMC3646728

[B24] Hollins M, Faldowski R, Rao S, Young F (1993) Perceptual dimensions of tactile surface texture: a multidimensional scaling analysis. Percept Psychophys 54:697–705. 10.3758/bf03211795 8134240

[B25] Hollins M, Bensmaïa S, Karlof K, Young F (2000) Individual differences in perceptual space for tactile textures: evidence from multidimensional scaling. Percept Psychophys 62:1534–1544. 10.3758/bf03212154 11140177

[B26] Howells J, Trevillion L, Bostock H, Burke D (2012) The voltage dependence of I(h) in human myelinated axons. J Physiol 590:1625–1640. 10.1113/jphysiol.2011.225573 22310314 PMC3413487

[B27] Hughes CL, Flesher SN, Weiss JM, Boninger ML, Collinger J, Gaunt R (2021) Perception of microstimulation frequency in human somatosensory cortex. Elife 10:e65128. 10.7554/eLife.65128 34313221 PMC8376245

[B28] Hursh JB (1939) Conduction velocity and diameter of nerve fibers. Am J Physiol 127:131–139. 10.1152/ajplegacy.1939.127.1.131

[B29] Johansson RS, Flanagan JR (2009) Coding and use of tactile signals from the fingertips in object manipulation tasks. Nat Rev Neurosci 10:345–359. 10.1038/nrn2621 19352402

[B30] Johansson RS, Vallbo AB (1979) Tactile sensibility in the human hand: relative and absolute densities of four types of mechanoreceptive units in glabrous skin. J Physiol 286:283–300. 10.1113/jphysiol.1979.sp012619 439026 PMC1281571

[B31] Johansson RS, Vallbo AB (1983) Tactile sensory coding in the glabrous skin of the human hand. Trends Neurosci 6:27–32. 10.1016/0166-2236(83)90011-5

[B32] Kaczmarek KA, Haase SJ (2003) Pattern identification and perceived stimulus quality as a function of stimulation waveform on a fingertip-scanned electrotactile display. IEEE Trans Neural Syst Rehabil Eng 11:9–16. 10.1109/TNSRE.2003.810421 12797720

[B33] LaMotte RH, Mountcastle VB (1975) Capacities of humans and monkeys to discriminate vibratory stimuli of different frequency and amplitude: a correlation between neural events and psychological measurements. J Neurophysiol 38:539–559. 10.1152/jn.1975.38.3.539 1127456

[B34] Lieber JD, Bensmaia SJ (2019) High-dimensional representation of texture in somatosensory cortex of primates. Proc Natl Acad Sci U S A 116:3268–3277. 10.1073/pnas.1818501116 30718436 PMC6386651

[B35] Macefield BYG, Gandevia SC, Burke D (1990) Perceptual responses to microstimulation of single afferents innervating joints, muscles, and skin of the human hand. J Physiol 429:113–129. 10.1113/jphysiol.1990.sp018247 2148951 PMC1181690

[B36] Mackevicius EL, Best MD, Saal HP, Bensmaia SJ (2012) Millisecond precision spike timing shapes tactile perception. J Neurosci 32:15309–15317. 10.1523/JNEUROSCI.2161-12.2012 23115169 PMC3752122

[B37] McGlone F, Reilly D (2010) The cutaneous sensory system. Neurosci Biobehav Rev 34:148–159. 10.1016/j.neubiorev.2009.08.004 19712693

[B38] McIntyre CC, Richardson AG, Grill WM (2002) Modeling the excitability of mammalian nerve fibers: influence of afterpotentials on the recovery cycle. J Neurophysiol 87:995–1006. 10.1152/jn.00353.2001 11826063

[B39] Mogyoros I, Kiernan MC, Burke D (1996) Strength-duration properties of human peripheral nerve. Brain 119:439–447. 10.1093/brain/119.2.439 8800939

[B40] Mountcastle VB, Talbot WH, Sakata H, Hyvärinen J (1969) Cortical neuronal mechanisms in flutter-vibration studied in unanesthetized monkeys. Neuronal periodicity and frequency discrimination. J Neurophysiol 32:452–484. 10.1152/jn.1969.32.3.452 4977839

[B41] Mountcastle VB, Steinmetz MA, Romo R (1990) Frequency discrimination in the sense of flutter: psychophysical measurements correlated with postcentral events in behaving monkeys. J Neurosci 10:3032–3044. 2118947 10.1523/JNEUROSCI.10-09-03032.1990 PMC6570255

[B42] Muniak MA, Ray S, Hsiao SS, Dammann JF, Bensmaia SJ (2007) The neural coding of stimulus intensity: linking the population response of mechanoreceptive afferents with psychophysical behavior. J Neurosci 27:11687–11699. 10.1523/JNEUROSCI.1486-07.2007 17959811 PMC6673240

[B43] Ng KKW, Olausson C, Vickery RM, Birznieks I (2020) Temporal patterns in electrical nerve stimulation: burst gap code shapes tactile frequency perception. PLoS One 15:e0237440. 10.1371/journal.pone.0237440 32790784 PMC7425972

[B44] Ng KKW, Snow IN, Birznieks I, Vickery RM (2021) Burst gap code predictions for tactile frequency are valid across the range of perceived frequencies attributed to two distinct tactile channels. J Neurophysiol 125:687–692. 10.1152/jn.00662.2020 33439792

[B45] Ochoa J, Torebjörk E (1983) Sensations evoked by intraneural microstimulation of single mechanoreceptor units innervating the human hand. J Physiol 342:633–654. 10.1113/jphysiol.1983.sp014873 6631752 PMC1193981

[B46] Oddo CM, Raspopovic S, Artoni F, Mazzoni A, Spigler G, Petrini F, Giambattistelli F, Vecchio F, Miraglia F, Zollo L, Di Pino G, Camboni D, Carrozza MC, Guglielmelli E, Rossini PM, Faraguna U, Micera S (2016) Intraneural stimulation elicits discrimination of textural features by artificial fingertip in intact and amputee humans. Elife 5:e09148. 10.7554/eLife.09148 26952132 PMC4798967

[B47] Ortiz-Catalan M, Håkansson B, Brånemark R (2014) An osseointegrated human-machine gateway for long-term sensory feedback and motor control of artificial limbs. Sci Transl Med 6:257re6. 10.1126/scitranslmed.3008933 25298322

[B48] Pei Y-C, Denchev PV, Hsiao SS, Craig JC, Bensmaia SJ (2009) Convergence of submodality-specific input onto neurons in primary somatosensory cortex. J Neurophysiol 102:1843–1853. 10.1152/jn.00235.2009 19535484 PMC2746774

[B49] Perovic M, Stevanovic M, Jevtic T, Strbac M, Bijelic G, Vucetic C, Popovic-Maneski L, Popovic D (2013) Electrical stimulation of the forearm: a method for transmitting sensory signals from the artificial hand to the brain. J Autom Control 21:13–18. 10.2298/JAC1301013P

[B50] Prsa M, Kilicel D, Nourizonoz A, Lee K-S, Huber D (2021) A common computational principle for vibrotactile pitch perception in mouse and human. Nat Commun 12:5336. 10.1038/s41467-021-25476-9 34504074 PMC8429766

[B51] Raspopovic S, Capogrosso M, Petrini FM, Bonizzato M, Rigosa J, Di Pino G, Carpaneto J, Controzzi M, Boretius T, Fernandez E, Granata G, Oddo CM, Citi L, Ciancio AL, Cipriani C, Carrozza MC, Jensen W, Guglielmelli E, Stieglitz T, Rossini PM, et al (2014) Restoring natural sensory feedback in real-time bidirectional hand prostheses. Sci Transl Med 6:222ra19. 10.1126/scitranslmed.3006820 24500407

[B52] Richardson AG, McIntyre CC, Grill WM (2000) Modelling the effects of electric fields on nerve fibres: influence of the myelin sheath. Med Biol Eng Comput 38:438–446. 10.1007/BF02345014 10984943

[B53] Roy EA, Hollins M (1998) A ratio code for vibrotactile pitch. Somatosens Mot Res 15:134–145. 10.1080/08990229870862 9730114

[B54] Saal HP, Bensmaia SJ (2014) Touch is a team effort: interplay of submodalities in cutaneous sensibility. Trends Neurosci 37:689–697. 10.1016/j.tins.2014.08.012 25257208

[B55] Saal HP, Harvey MA, Bensmaia SJ (2015) Rate and timing of cortical responses driven by separate sensory channels. Elife 4:e10450. 10.7554/eLife.10450 26650354 PMC4755746

[B56] Schady WJ, Torebjörk HE, Ochoa JL (1983) Peripheral projections of nerve fibres in the human median nerve. Brain Res 277:249–261. 10.1016/0006-8993(83)90932-0 6640299

[B57] Sur M, Wall JT, Kaas JH (1981) Modular segregation of functional cell classes within the postcentral somatosensory cortex of monkeys. Science 212:1059–1061. 10.1126/science.7233199 7233199

[B58] Sur M, Wall JT, Kaas JH (1984) Modular distribution of neurons with slowly adapting and rapidly adapting responses in area 3b somatosensory cortex in monkeys. J Neurophysiol 51:724–744. 10.1152/jn.1984.51.4.724 6716121

[B59] Szeto AYJ, Lyman J, Prior RE (1979) Electrocutaneous pulse rate and pulse width psychometric functions for sensory communications. Hum Factors 21:241–249. 10.1177/001872087902100212 489023

[B60] Talbot WH, Darian-Smith I, Kornhuber HH, Mountcastle VB (1968) The sense of flutter-vibration: comparison of the human capacity with response patterns of mechanoreceptive afferents from the monkey hand. J Neurophysiol 31:301–334. 10.1152/jn.1968.31.2.301 4972033

[B61] Tan DW, Schiefer MA, Keith MW, Anderson JR, Tyler J, Tyler DJ (2014) A neural interface provides long-term stable natural touch perception. Sci Transl Med 6:257ra138. 10.1126/scitranslmed.3008669 PMC5517305 25298320

[B62] Tashiro T, Higashiyama A (1981) The perceptual properties of electrocutaneous stimulation: sensory quality, subjective intensity, and intensity-duration relation. Percept Psychophys 30:579–586. 10.3758/bf03202013 7335455

[B63] Torebjörk HE, Vallbo AB, Ochoa JL (1987) Intraneural microstimulation in man: its relation to specificity of tactile sensations. Brain 110:1509–1529. 10.1093/brain/110.6.1509 3322500

[B64] Valle G, Mazzoni A, Iberite F, D'Anna E, Strauss I, Granata G, Controzzi M, Clemente F, Rognini G, Cipriani C, Stieglitz T, Petrini FM, Rossini PM, Micera S (2018) Biomimetic intraneural sensory feedback enhances sensation naturalness, tactile sensitivity, and manual dexterity in a bidirectional prosthesis. Neuron 100:37–45.e7. 10.1016/j.neuron.2018.08.033 30244887

[B65] Verrillo RT, Fraioli AJ, Smith RL (1969) Sensation magnitude of vibrotactile stimuli. Percept Psychophys 6:366–372. 10.3758/BF03212793

[B66] Weber AI, Saal HP, Lieber JD, Cheng J-W, Manfredi LR, Dammann JF, Bensmaia SJ (2013) Spatial and temporal codes mediate the tactile perception of natural textures. Proc Natl Acad Sci U S A 110:17107–17112. 10.1073/pnas.1305509110 24082087 PMC3800989

[B67] Wu G, Ekedahl R, Stark B, Carlstedt T, Nilsson B, Hallin RG (1999) Clustering of Pacinian corpuscle afferent fibres in the human median nerve. Exp Brain Res 126:399–409. 10.1007/s002210050746 10382624

[B68] Yau JM, Hollins M, Bensmaia SJ (2009) Textural timbre: the perception of surface microtexture depends in part on multimodal spectral cues. Commun Integr Biol 2:344–346. 10.4161/cib.2.4.8551 19721886 PMC2734043

